# The DEK Oncogene Is a Target of Steroid Hormone Receptor Signaling in Breast Cancer

**DOI:** 10.1371/journal.pone.0046985

**Published:** 2012-10-10

**Authors:** Lisa M. Privette Vinnedge, Shuk-Mei Ho, Kathryn A. Wikenheiser-Brokamp, Susanne I. Wells

**Affiliations:** 1 Cancer and Blood Diseases Institute, Cincinnati Children’s Hospital Medical Center, Cincinnati, Ohio, United States of America; 2 Department of Environmental Health, University of Cincinnati College of Medicine and Cincinnati Veteran Affairs Medical Center, Cincinnati, Ohio, United States of America; 3 Department of Pathology and Laboratory Medicine, Cincinnati Children’s Hospital Medical Center and University of Cincinnati College of Medicine, Cincinnati, Ohio, United States of America; II Università di Napoli, Italy

## Abstract

Expression of estrogen and progesterone hormone receptors indicates a favorable prognosis due to the successful use of hormonal therapies such as tamoxifen and aromatase inhibitors. Unfortunately, 15–20% of patients will experience breast cancer recurrence despite continued use of tamoxifen. Drug resistance to hormonal therapies is of great clinical concern so it is imperative to identify novel molecular factors that contribute to tumorigenesis in hormone receptor positive cancers and/or mediate drug sensitivity. The hope is that targeted therapies, in combination with hormonal therapies, will improve survival and prevent recurrence. We have previously shown that the DEK oncogene, which is a chromatin remodeling protein, supports breast cancer cell proliferation, invasion and the maintenance of the breast cancer stem cell population. In this report, we demonstrate that DEK expression is associated with positive hormone receptor status in primary breast cancers and is up-regulated *in vitro* following exposure to the hormones estrogen, progesterone, and androgen. Chromatin immunoprecipitation experiments identify *DEK* as a novel estrogen receptor α (ERα) target gene whose expression promotes estrogen-induced proliferation. Finally, we report for the first time that DEK depletion enhances tamoxifen-induced cell death in ER+ breast cancer cell lines. Together, our data suggest that DEK promotes the pathogenesis of ER+ breast cancer and that the targeted inhibition of DEK may enhance the efficacy of conventional hormone therapies.

## Introduction

Clinical and pathological characterization of breast cancer, the second leading cause of cancer-related deaths among women in the United States [1], is crucial for identifying the best course of treatment for each patient. Detection of steroid hormone receptor expression, particularly estrogen, progesterone, and androgen receptors, determines whether or not a patient will respond to selective estrogen receptor (ER) modulators (SERMS), aromatase inhibitors, or other anti-hormone therapies. In recent decades, there has been an increase in the percentage of breast cancers that are positive for the expression of ERα (“ER+”) such that nearly 75% of all breast cancers are now ER+ [2]. Continued advancements in our understanding of the biology of these cancers are important in order to generate novel, more effective and perhaps combinatorial treatments.

Recent reports have shown that transcription of the *DEK* oncogene is up-regulated in breast cancers with particularly strong gene expression detected in lymph node positive and late stage breast cancers, and that DEK expression correlated with increased recurrence rates after 3 years [3]–[6]. Furthermore, work from our laboratory has shown that DEK protein levels are elevated in both cultured cell lines and primary invasive adenocarcinomas and that DEK expression stimulates breast cancer cell proliferation *in vitro* and *in vivo,* together with cellular invasion, and growth of the breast cancer stem cell population [7].

DEK is a unique, ubiquitously expressed protein that predominantly binds to chromatin but can also be soluble or secreted as a result of post-translational modifications [8]–[12]. Its ability to bind nucleic acids has led to functional associations with several cellular processes including chromatin remodeling, transcriptional regulation, replication, mRNA splicing, and DNA repair [13]–[19]. Cell free assays have shown that DEK introduces constrained positive supercoils into DNA and can facilitate the ligation of linear DNA molecules *in vitro* [20], [21]. Importantly, DEK plays a critical role in chromatin organization and the maintenance of genome stability *in vivo*. It has histone chaperone activity, which regulates epigenetic markers on chromatin, and it is necessary for the maintenance of heterochromatin integrity by facilitating the interaction between Heterochromatin Protein 1α (HP1α) and trimethylated histone H3 (H3K9me3) [22], [23]. These effects on chromatin structure are likely responsible for alterations in gene transcription and DNA replication by regulating accessibility to DNA. In addition, DEK recently was found to promote DNA-PK activity and DNA double-strand break repair by non-homologous end joining (NHEJ) [19]. Therefore, when DEK is up-regulated, as is observed in numerous types of cancer including breast cancer, perturbations to normal genome architecture and integrity are likely contributors to oncogenesis [3], [4], [7], [24]–[28]. In addition to epigenetics and chromatin integrity, the DEK oncogene has also been implicated in regulating the expression, phosphorylation and/or activity of several important signaling molecules and pathways. For example, DEK enhances invasion in breast cancer cell lines by stimulating β-catenin activity [7]. Furthermore, DEK inhibits p53-dependent and –independent apoptosis and has been found to mediate the apoptotic response to clastogenic chemotherapeutic agents such as doxorubicin and cisplatin [7], [28]–[30].

It is well documented that the levels of DEK protein are critical for events such as oncogenesis and genome stability. The over-expression of DEK stimulated tumorigenesis in several tissues, and DEK depletion can result in cell death and impaired DNA double strand break repair [7], [19]. Therefore, cellular DEK expression is tightly controlled in order to maintain proper cell function and viability. However, little is known about cellular regulation of *DEK* gene expression. NF-Y and YY1 were shown to be responsible for the constitutive transcription of *DEK* and the *DEK-CAN* fusion gene (found in t(6;9) acute myeloid leukemias) in transformed cell lines [31]. DEK is also an E2F target gene and consequently up-regulated in cells infected with human papillomavirus (HPV) due to inactivation of Rb and the subsequent activation of E2F transcription factors [27]. Here, we report for the first time that *DEK* transcription is regulated by steroid hormone receptors, particularly ERα in breast cancer, and that DEK expression promotes hormone-dependent cancer cell proliferation.

## Results

### Hormone Receptor Positive Primary Breast Cancers Express the DEK Oncogene

**Figure 1:**
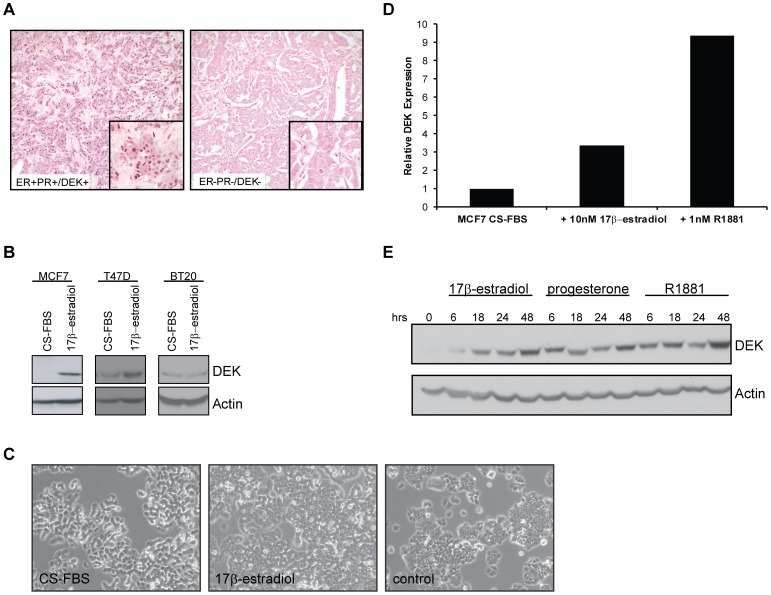
DEK expression is associated with positive hormone receptor status in human primary and cultured breast cancers. (A) Estrogen receptor (ER) negative tumors were often negative for DEK staining (right) while ER positive tumors were often positive for DEK staining (left). Immunohistochemical staining for DEK in two invasive ductal carcincomas showing positive DEK staining (DEK+) in a hormone receptor positive (ER+/PR+) tumor and lack of DEK expression (DEK-) in a hormone receptor negative tumor (ER−/PR−). Low power images are at 200× and all high power images are at 1000× magnification. (B) Western blotting for DEK showed increased expression following exposure to 17β-estradiol for 48 hours in ER+ MCF7 and T47D cells but not in ER- BT20 cells. (C) Cell morphology of T47D cells in CS-FBS (left), 48 hours of 17β-estradiol treatment (middle), and under normal culture conditions (right). Bright field images of cultured cells were obtained at 100× total magnification. (D) *DEK* expression increases in 17β-estradiol and R1881 treated cells. Quantitative RT-PCR was performed to detect *DEK* expression in hormone starved MCF7 cells treated with 10 nM 17β-estradiol or 1 µM methyltrienolone (R1881) for six hours. *GAPDH* was used as a control and values are normalized to the untreated sample. (E) Western blotting for DEK shows increased protein levels after treatment of hormone starved MCF7 cells with 10 nM 17β-estradiol, 10 nM progesterone, or 1 µM methyltrienolone (R1881) over the course of 48 hours.

We performed immunohistochemical analysis for DEK expression on a tissue microarray that consisted of 30 invasive breast carcinomas and compared expression levels with numerous clinical and pathological variables including patient age, tumor grade, tumor stage, tumor size, lymph node status, HER2 expression, and hormone receptor status. Of those, possible associations between positive DEK expression and both androgen receptor (AR) positivity and patient age greater than 50 years old manifested as a trend. However, there was a strong positive relationship between DEK expression and progesterone and estrogen hormone receptor positive primary invasive breast adenocarcinomas (Table 1; Fig. 1A). This contradicts a recent report by Liu *et al*. which did not find a correlation between DEK and ER/PR expression in invasive adenocarcinomas [6]. Besides using a different patient population, the major difference between our work and theirs is that they only considered cancers that were “strongly positive” for DEK expression; here, we uncovered an association between ER/PR/AR positivity with any degree of DEK staining.

**Table 1:** Association of DEK expression with clinical and pathological variables in invasive adenocarcinomas of the breast.

Clinical & Pathological Variables	N	Number DEK+	p value*
Age			
≤50 yr	21	15	
>50 yr	9	9	0.07
Estrogen Receptor			
Negative	19	13	
Positive	11	11	0.04
Progesterone Receptor			
Negative	20	14	
Positive	10	10	0.05
Androgen Receptor			
Negative	21	15	
Positive	9	9	0.07

* Chi-Squared statistic.

### DEK is an Estrogen Receptor Target Gene

In order to determine if DEK expression was associated with hormone receptor expression and activity *in vitro,* two ER+/PR+/AR+ cell lines, MCF7 and T47D, and ER−/PR−/AR− BT20 cells were cultured in hormone depleted charcoal-stripped serum (CS-FBS) then treated with 10 nM 17β-estradiol (E2) to activate the estrogen receptor. DEK expression was significantly up-regulated in the two ER+ cell lines, particularly in MCF7 cells, upon 17β-estradiol exposure but was unchanged in ER− BT20 cells (Fig. 1B). In addition, 17β-estradiol exposure of T47D cells restored the epithelial morphology and induced proliferation (Fig. 1C). Efficacy of 17β-estradiol treatment was confirmed by western blotting for phosphorylated p44/42 (Erk1/2; Figure S1). Further analysis revealed that all three hormones, 17β-estradiol, progesterone, and the synthetic androgen R1881, resulted in increased expression of DEK mRNA and/or protein as early as 6 hours after treatment (Fig. 1D and 1E). Based on the strength of the association between DEK expression and ER status in primary adenocarcinomas (p = 0.04), we decided to focus on the mechanism of 17β-estradiol mediated DEK up-regulation.

**Figure 2:**
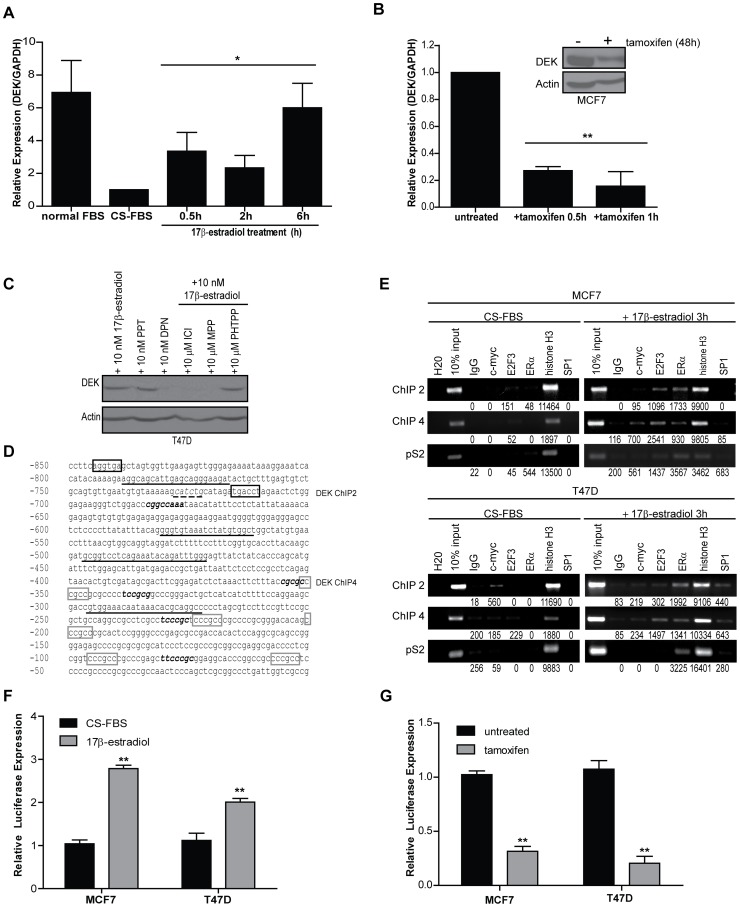
ERα binds to the *DEK* promoter in response to 17β-estradiol exposure and receptor activity correlates with *DEK* expression. (A) 17β-estradiol treatment causes a rapid 4-fold increase in *DEK* transcription. Quantitative RT-PCR was performed to detect *DEK* expression in hormone starved MCF7 cells treated with 10 nM 17β-estradiol during the time course shown. *GAPDH* was used as a control and values are normalized to the untreated sample and represented as fold-change. Results shown depict the average of two to five independent experiments. An asterisk (*) indicates p≤0.05 as determined by one-way ANOVA. (B) Treatment with 3 µg/ml tamoxifen results in a dramatic decrease in *DEK* expression. Quantitative RT-PCR was performed to detect *DEK* expression in MCF7 cells grown in low serum treated with 3 µg/ml tamoxifien during the time course shown. *GAPDH* was used as a control and values are normalized to the untreated sample and represent fold change. Results shown depict the average of replicate experiments. Two asterisks (**) indicates p≤0.01 as determined by one-way ANOVA. (C) ERα, not ERβ, stimulates 17β-estradiol induced DEK expression as determined by western blot analysis. T47D cells were grown in CS-FBS for 7 days then treated with either 10 nM of agonists or 10 µM of antagonist in the presence of 10 nM 17β-estradiol. (D) *DEK* gene promoter −850 bp upstream of the 5′ UTR. Primers used for ChIP are depicted by underlined sequences. The ERE half sites, described as “DEK ChIP 2,” are highlighted in black boxes at positions −845 and −716 bp. The putative ERα/SP1 binding sites are highlighted with gray boxes drawn starting at position −352 and were amplified as “DEK ChIP 4.” E2F and E2F3 binding sites, previously characterized by Carro *et al* [27], are indicated by bold, italicized text. The putative c-myc binding site is underlined with a dashed line. (E) ERα binds to the *DEK* promoter in response to 17β-estradiol treatment. Chromatin was isolated from MCF7 and T47D cells cultured in CS-FBS that were either untreated or treated for 3 hours with 10 nM 17β-estradiol. Chromatin was then subjected to immunoprecipitation using antibodies for IgG (negative control), c-myc, E2F3, ERα, histone H3 (positive control), or SP1. “Input” represents 10% of the DNA used in the immunoprecipitation. Two loci were tested in the *DEK* promoter, “DEK ChIP 2″ and “DEK ChIP 4″ (see above). The *pS2* gene promoter was used as a positive control for ERα binding following 17β-estradiol treatment. Raw densitometry values are indicated under the gel images. (F) *DEK* reporter assays show transcriptional up-regulation in response to 17β-estradiol treatment. MCF7 and T47D cells were transfected with a luciferase reporter construct under the control of a 1200 bp fragment of the *DEK* promoter and the first exon. Cells were treated with 10 nM 17β-estradiol for 24 hours. Data represent the average fold induction of luciferase expression above untreated (CS-FBS) levels from triplicate experiments. Two asterisks (**) indicate p≤0.01 as determined by Student’s t-test. (G) DEK reporter assays show transcriptional down-regulation in response to tamoxifen treatment. MCF7 and T47D cells were transfected with a luciferase reporter construct as in (F). Cells were treated with tamoxifen for 24 hours. Data represent the average fold reduction of luciferase expression below untreated levels from triplicate experiments. Two asterisks (**) indicate p≤0.01 as determined by Student’s t-test.

Analysis of microarray data in Oncomine indicated that at least 14 independent studies showed a correlation between *DEK* mRNA expression with estrogen receptor status such that *DEK* expression was lower in primary ER+ breast cancers compared to ER- breast cancers. In contrast, we showed high levels of DEK protein in ER+ breast cancers (Fig. 1). We were thus intrigued by a report by Coser *et al.* that revealed *DEK* mRNA levels increased in MCF7/BUS cells treated with 17β-estradiol in a dose-dependent manner [5], [32]. We also have observed that DEK protein levels were up-regulated in T47D cells at these minimal doses (Figure S2). To determine how rapidly *DEK* expression increases after 17β-estradiol treatment, MCF7 cells were cultured in CS-FBS and then exposed to 10 nM 17β-estradiol for 30 minutes, 2 hours, and 6 hours and analyzed by quantitative RT-PCR. A nearly four-fold increase in *DEK* expression was observed at 30 minutes post-treatment and remained high up until the last time point (Fig. 2A). Prolonged exposure to 17β-estradiol resulted in *DEK* transcript expression returning to baseline levels by 24 hours (Figure S3). Despite the transient mRNA induction, DEK protein levels increased further even after 48 hours of 17β-estradiol treatment (Figure 1E).

Activity of the estrogen receptor can be chemically inhibited by a family of drugs called selective estrogen receptor modulators (SERMs), an example of which is the commonly used chemotherapeutic drug tamoxifen. MCF7 cells treated with 3 µg/ml tamoxifen rapidly down-regulated *DEK* expression as determined by quantitative RT-PCR (Fig. 2B). This translated into substantially decreased levels of DEK protein as detected by western blotting after 48 hours of treatment with tamoxifen (Fig. 2B, inset).

In order to ascertain if ERα or ERβ activation was responsible for 17β-estradiol mediated DEK expression, we used a combination of ER agonists and antagonists that differentially altered the activity of the two receptors. Treatment with 17β-estradiol (ERα/β agonist) and propylpyrazole triol (PPT; ERα agonist) both stimulated DEK expression whereas diarylpropionitrile (DPN; ERβ agonist) did not. Likewise, treatment with ICI-182780 (fulvestrant; ERα/β antagonist) and methyl-piperidino-pyrazole (MPP; ERα antagonist) inhibited 17β-estradiol induced DEK expression whereas pyrazolo [1,5-a] pyrimidine (PHTPP; ERβ antagonist) did not (Fig. 2C). Collectively, this indicates that ERα, not ERβ, is responsible for DEK up-regulation following exposure to 17β-estradiol.

We next determined whether 17β-estradiol could induce ERα binding to the *DEK* promoter. *In silico* analysis using both the Pattern Search for Transcription Factor Binding Sites (PATCH) and Transcription Element Search Software (TESS) software programs identified a cluster of putative ERα/SP1 binding sites, which are non-canonical ERα binding sites, within 1 kb of the transcriptional start site of the *DEK* gene (Fig. 2D, gray boxes) and both AR and PR binding sites within 3 kb of the transcriptional start site and within the first intron (data not shown) [33]–[36]. Additional searches revealed a canonical estrogen response element (ERE) approximately 800 bp upstream of the transcriptional start site (Fig. 2D, half sites indicated by black boxes). To determine if ERα can bind directly to select sites in the *DEK* promoter, we performed chromatin immunoprecipitation (ChIP). Briefly, chromatin was collected from hormone starved and 17β-estradiol treated MCF7 and T47D cells and subjected to immunoprecipitation with antibodies for IgG (negative control), histone H3 (positive control), the known *DEK* regulator E2F3, the β-catenin target c-myc, ERα, and SP1 [27]. We attempted to detect the multiple ERα/SP1 sites in the first 250 bp of the *DEK* promoter but the GC-rich region was technically difficult to amplify. Two other locations were tested within the *DEK* promoter (ChIP 2 and ChIP 4) as well as the promoter of the pS2 gene, a known ERα target gene. First, we determined that c-myc can bind the *DEK* promoter in starved T47D cells and low levels of E2F3 can bind the *DEK* promoter in hormone starved MCF7 cells (Fig. 2E, left panels). This relatively high degree of c-myc binding may explain why the baseline level of DEK in T47D cells grown in CS-FBS was higher with a modest increase in DEK expression following 17β-estradiol treatment compared to MCF7 cells (Fig. 1B). None of the other transcription factors were bound to the *DEK* promoter under conditions of hormone starvation. Interestingly, ERα was detected at both loci in the *DEK* promoter in the presence of 17β-estradiol, possibly in a complex with SP1. This suggests that *DEK* is an ERα target gene, which is up-regulated rapidly after exposure to 17β-estradiol. Luciferase reporter assays confirmed that transcription from the *DEK* promoter was up-regulated following 17β-estradiol treatment (Fig. 2F) and down-regulated in response to tamoxifen-mediated inhibition of estrogen receptor activity (Fig. 2G). Together, this suggests that the regions amplified in ChIP 2 and 4 contain putative binding sites for ERα and there may be additional sites outside of these regions that result in steroid hormone induced up-regulation of *DEK* oncogene expression. Therefore, detailed mutational studies will be critical to uncover their functional importance, and that of associated transcription factor complexes, in human breast cancer cells and cell lines.

Furthermore, the Wnt/β-catenin target c-myc also bound to the *DEK* promoter in the region of ChIP4 and E2F3 bound to both ChIP2 and ChIP4, as well as on the pS2 promoter following treatment with 17β-estradiol. Given the concomitant binding of the E2F3 and c-myc transcription factors that are both pro-proliferative with ERα binding, we hypothesized that DEK promotes cellular proliferation following 17β-estradiol exposure.

### DEK is Necessary for 17β-estradiol-stimulated Cell Proliferation

**Figure 3:**
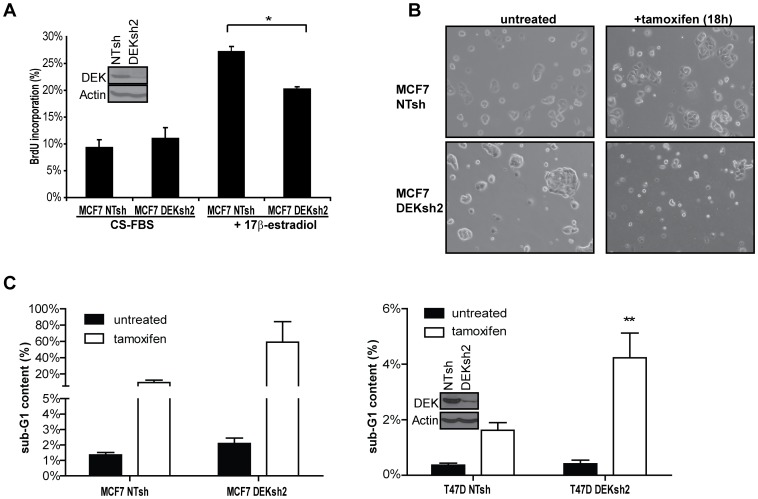
DEK is necessary for 17β-estradiol stimulated cell proliferation and modulates sensitivity to tamoxifen. (A) DEK expression is required for 17β-estradiol stimulated cellular proliferation. Hormone starved MCF7 cells transduced with non-targeting shRNA (NTsh) or DEK shRNA (DEKsh2) were untreated (CS-FBS) or exposed to 10 nM 17β-estradiol, then cultured in BrdU. The percentage of BrdU positive cells was determined by flow cytometry. Asterisk (*) denotes p<0.05 using Student’s t-test. (B and C) DEK depletion by shRNA (DEKsh2) works synergistically with tamoxifen to induce apoptosis in breast cancer cell lines. (B) Bright field images (100× magnification) of MCF7 cells expressing either NTsh or DEKsh2 were cultured in low serum media and either untreated or treated with tamoxifen for 18 hours. (C) DEK depletion by shRNA (DEKsh2) enhances the cytotoxic effect of tamoxifen. DEK proficient and deficient MCF7 (left) and T47D (right) cells were grown in low serum media then treated with 3 µg/ml tamoxifen for 22 hours. Cells were labeled with 7AAD then analyzed for sub-G1 content by flow cytometry as a measure of apoptosis. Results shown are the average of triplicate experiments. Two asterisks (**) indicate p<0.01 as determined using a 2-way ANOVA test for significance. For MCF7 cells, p = 0.08. (A and B insets) DEK shRNA knockdown is shown by western blot analysis for normally cultured cells that were transduced with lentivirus carrying either non-targeting shRNA (NTsh) or DEK specific shRNA (DEKsh2).

Estrogen is a well-characterized mitogenic signal that stimulates cellular proliferation in primary and cultured breast cancer cells, including the MCF7 cell line [37]–[39]. The DEK oncogene also has been implicated in proliferation and survival in several different cell and tissue types in the absence of estrogen modulation [30], [40]. To determine if the up-regulation of DEK in response to 17β-estradiol was functionally relevant for breast cancer pathogenesis, MCF7 cells were infected with lentiviruses carrying non-targeting shRNA (NTsh) or DEKshRNA (DEKsh2) as previously described (Fig. 3A inset) [7]. After selection with puromycin, the cells were plated in CS-FBS media with or without 10 nM 17β-estradiol, and BrdU incorporation was monitored as a measure of DNA replication. There was no difference in BrdU incorporation between the two hormone starved cell lines. However, DEK knockdown resulted in a significant decrease in the proliferative response to 17β-estradiol compared to control NTsh cells (Fig. 3A, p<0.05). Taken together, this data demonstrates that DEK is necessary for hormone-stimulated proliferation in the ER+ MCF7 breast cancer cell line.

### Loss of DEK Sensitizes ER+ Breast Cancer Cells to Tamoxifen

The estrogen receptor antagonist tamoxifen is used therapeutically to inhibit proliferation and promote apoptosis in ER+ breast cancers. Tamoxifen treatment decreased DEK expression (Fig. 2B), but DEK was still expressed to detectable levels. To determine if the remaining DEK protein in tamoxifen treated cells was important for the cellular response to therapy, control and DEK-depleted (DEKsh2) cells were treated with tamoxifen and analyzed for apoptosis, as detected by sub-G1 DNA content using flow cytometry and visual analysis with bright field microscopy. As previously reported, loss of DEK by itself increased the percentage of apoptotic cells in the population (Fig. 3C) [7]. In addition to this baseline increase in apoptosis, DEK depletion by shRNA enhanced the apoptotic response to tamoxifen treatment in MCF7 and T47D cells (Fig. 3B, 3C). These findings were confirmed by DAPI staining to visualize condensed and fragmented DNA in apoptotic cells that remained attached to the culture surface, and western blotting indicated the presence of increased levels of cleaved caspase 8 (Fig. S4). Combined, these data suggest that the two methodologies, DEK depletion and tamoxifen treatment, can act synergistically to eliminate ER+ cancer cells in culture.

## Discussion

Herein we report for the first time a functional association between 17β-estradiol exposure and DEK expression in ER+ breast cancers and identify *DEK* as a novel ERα target gene. DEK expression was associated with the presence of steroid hormone receptors in primary tumors and its expression was upregulated in response to steroid hormone treatment, with a particular focus on 17β-estradiol in ER+ MCF7 and T47D cells *in vitro*. *DEK* transcription was upregulated rapidly, within 30 minutes of 17β-estradiol treatment, but then fell to baseline levels with prolonged exposure (Figures 2 and S3). This transient synthesis of *DEK* message may explain why microarray studies in Oncomine, using tissues persistently exposed to estrogen, have typically reported lower DEK expression in ER+ cancers when compared to ER- cancers. Prolonged DEK up-regulation at the protein, but not transcript, level therefore also suggests post-transcriptional regulatory mechanisms following hormone exposure, but the regulation of DEK protein stability and turnover in breast cancer cells is unknown. DEK induction by 17β-estradiol signaling also was functionally important since the RNAi-mediated loss of DEK expression in ER+ MCF7 cells diminished 17β-estradiol induced cell proliferation. Together, our work suggests that, *in vivo,* hormone signaling, such as 17β-estradiol exposure, might result in the up-regulation of DEK protein levels to promote proliferation in ER+ cancers.

**Figure 4:**
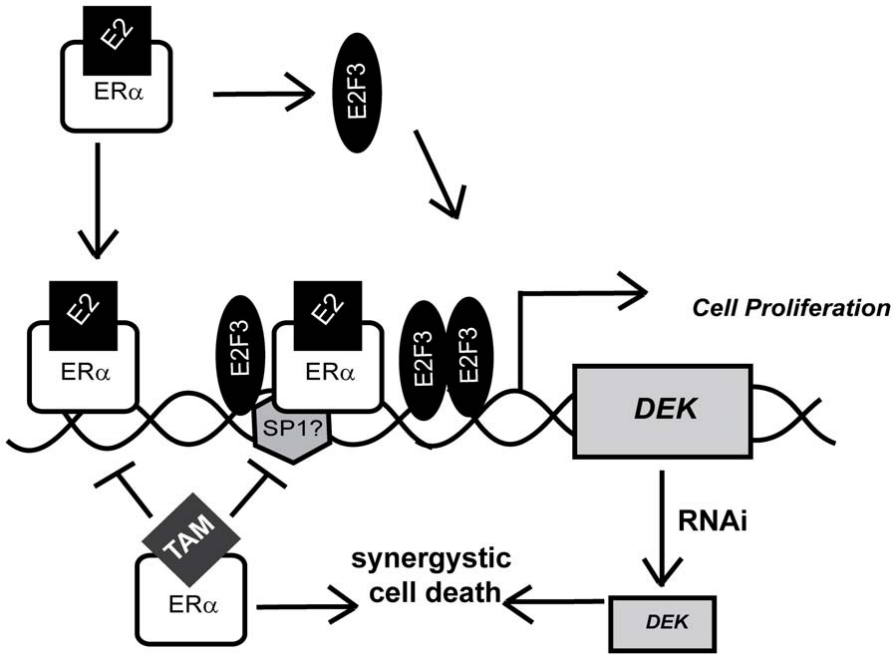
Model for DEK transcriptional up-regulation following 17β-estradiol exposure. Upon 17β-estradiol exposure, ERα is activated and binds to the *DEK* promoter at least at two locations – an ERE half site at −716 bp and at ERα/Sp1 binding sites more proximal to the transcriptional start site. A second potential mechanism of up-regulation is the ERα-mediated up-regulation of E2F proteins (particularly E2F3) that also increase *DEK* transcription. Increased levels of DEK then promote proliferation. DEK expression can be targeted with the anti-estrogen tamoxifen to inhibit cell proliferation. The knockdown of DEK by RNAi can increase tamoxifen sensitivity of ER+ cell lines by synergistically inducing an apoptotic response.

DEK expression was also observed in several primary ER- breast cancers and was previously published to be highly expressed in ER- breast cancer cell lines compared to immortalized mammary epithelial cells [7]. This may be because *DEK* is a known E2F target gene and thus upregulated upon Rb pathway deregulation, a frequent event in breast cancer [27], [41], [42]. E2F3 also was observed to bind the *DEK* promoter in response to 17β-estradiol treatment, but this binding was minimal in the growth-restricting CS-FBS containing media (Fig. 2E). Of note, E2F is a known ERα target gene; therefore, more than one mechanism may contribute to *DEK* up-regulation following the mitogenic signal of steroid hormone exposure [43]. First, ERα binding to the *DEK* promoter could directly enhance transcription. Second, activated ERα could drive the expression of E2F proteins, the binding of which to the *DEK* promoter might then further increase expression in an additive or synergistic fashion (Fig. 4). We postulate that DEK is transcriptionally up-regulated via ER-dependent and -independent mechanisms in a majority of breast cancers, and studies to dissect the relative contributions of E2F and ERα are currently under way. Importantly, the up-regulation of DEK in ER+ and ER- cancers suggest that targeting DEK expression may be a therapeutic option for breast cancers with different molecular signatures.

Previous reports have linked the role of DEK in DNA repair to resistance to clastogenic agents such as hydroxyurea, etoposide, doxorubicin, camptothecin, and neocarzinostatin [11], [19], [28], [44]. We report here, for the first time, that DEK depletion can also act synergistically with another class of chemotherapeutic drugs, SERMs, whose activity is unrelated to the DNA damage response. DEK depletion using RNAi enhanced the cytotoxic activity of tamoxifen in ER+ cell lines. Due to its ability to modulate apoptotic responses to multiple classes of drugs, chemical- or RNAi-based therapeutic DEK targeting approaches in cancer cells could substantially enhance the efficacy of existing therapies. Importantly, DEK depletion by RNAi has been shown to induce a dramatic cytotoxic effect in cancer cells but has relatively little, and no, toxicity in normal and differentiated cells, respectively [4], [30]. Therefore, DEK targeting combined with tamoxifen may be a promising method to enhance drug efficacy or to overcome drug resistance in ER+ breast cancers with limited effects on adjacent normal epithelia.

## Methods

### Cell Culture

Cell lines were obtained from the American Type Culture Collection (ATCC) and cultured as directed by the ATCC. For hormone responsiveness, cells were starved in media without phenol red or insulin and with 10% charcoal-stripped FBS (CS-FBS) for seven days prior to treatment with 10 nM 17β-estradiol (Sigma-Aldrich, St. Louis, MO, USA), 10 nM progesterone (Sigma-Aldrich, St. Louis, MO), 1 µM methyltrienolone (R1881; Sigma-Aldrich, St. Louis, MO), 10 nM propylpyrazole triol (PPT; Tocris Bioscience, Bristol, United Kingdom), 10 nM diarylpropionitrile (DPN; Tocris Bioscience, Bristol, United Kingdom), 10 µM ICI-182780 (ICI; Tocris Bioscience, Bristol, United Kingdom), 10 µM methyl-piperidino-pyrazole (MPP; Tocris Bioscience, Bristol, United Kingdom), and 10 µM pyrazolo [1,5-a] pyrimidine (PHTPP; Tocris Bioscience, Bristol, United Kingdom). For treatment with 3 µg/ml tamoxifen (Sigma-Aldrich, St. Louis, MO), cells were cultured as directed by the ATCC with the exception of 5% FBS instead of 10% FBS. Images were obtained with a Leica DMIL microscope (Leica Microsystems, Bannockburn, IL) and SPOT imaging software (Diagnostic Instruments, Sterling Heights, MI).

### Tissue Microarray

A breast tissue microarray (BRC961C_F, Pantomics, Inc., Richmond, CA) was stained by immunohistochemistry using the M.O.M. kit (M.O.M. Peroxidase Kit; Vector Laboratories, Burlingame, CA) and monoclonal DEK antibody (1∶60, BD Biosciences, San Jose, CA). The tissue microarray contained intact duplicate samples including normal breast (n = 2), invasive adenocarcinomas (n = 30, 28 ductal, one ductal papillary and one mucinous) and non-malignant breast disease including hyperplasia (n = 3), fibrocystic changes (n = 4) and fibroadenomas (n = 3). All patients were female with the exception of the one case of ductal papillary adenocarcinoma. Due to sample size and histological variability, we focused on the invasive adenocarcinomas for characterization. Breast cancer risk increases with age; the age of 50 years old was chosen in this study to (1) attempt to delineate between inherited breast cancers and sporadic breast cancers and (2) because overall risk and incidence increases after age 50 [45]. DEK staining was blindly scored as positive or negative based on the presence of any brown-stained nuclei. Tumors considered positive for hormone receptors showed staining for the receptor in ≥5% of cells.

### Western Blotting

Western blotting was performed as previously described [30], [46]. Membranes were probed with antibodies to DEK (1∶1000, BD Biosciences, San Jose, CA), phospho-p44/42 (1∶2000, Cell Signaling, Danvers, MA), total p44/42 (1∶2000, Cell Signaling, Danvers, MA), caspase 8 (1∶1000, Cell Signaling, Danvers MA), or Actin (1∶10,000; gift of James Lessard, Cincinnati Children’s Hospital Medical Center, Cincinnati, OH).

### Quantitative RT-PCR

Total mRNA was isolated, reverse transcribed and cDNA was amplified with SYBR Green PCR master mix using an ABI-7300 quantitative PCR machine (Applied Biosystems, Carlsbad, CA). The following forward (F) and reverse (R) primer pairs were used at a concentration of 0.4 ng/µl each: GAPDH F-(5′-GGTCTCCTCTGACTTCAACA) R-(5′-ATACCAGGAAATGAGCTTGA), and DEK F-(5′-TGTTAAGAAAGCAGATAGCAGCACC-3′) R-(5′-ATTAAAGGTTCATCATCTGAACTATCCTC-3′).

### Chromatin Immunoprecipitation (ChIP)

Chromatin immunoprecipitation was performed as previously described [47]. Briefly, MCF7 and T47D cells cultured in CS-FBS media were treated with 10 nM 17β-estradiol for 3 hours then crosslinked with formaldehyde for 10 minutes. Crosslinking was reversed by adding glycine to a final concentration of 0.125 M for 10 minutes. Cells were harvested, washed in PBS with protease inhibitors and resuspended in cell lysis buffer (5 mM PIPES (pH 8.0), 85 mM KCl, 0.5% Nonidet P-40, and protease inhibitors) for 30 min and centrifuged. Nuclei were isolated via centrifugation and resuspended in nuclei lysis buffer (50 mm Tris-Cl (pH 8.1), 10 mm EDTA, 1% SDS, and protease inhibitors) for 30 min. The chromatin was sonicated three times, for 10 seconds each time, to generate DNA fragments with a range of 100–1000 bp. Ten percent of the chromatin was kept as an input, and 70 µg was used for immuneprecipitation in combination with 1 µg of antibody and RIPA lysis buffer with protease inhibitors to a final volume of 500 µl. After three hours at 4°C, 20 µl of protein A slurry was added overnight. IPs were washed once in RIPA with 125 mM NaCl then three times with RIPA + protease inhibitors and two times with TE before incubating beads with extraction buffer (0.1 m NaHCO_3_, 1% SDS, 0.3 m NaCl, 10 mg/ml RNase A) at 65°C overnight to de-crosslink. Immunoprecipitated DNA was purified using the PCR purification kit (Qiagen, Valencia, CA) and resuspended in 50 µl of sterile water. The purified DNA was PCR-amplified and run on ethidium bromide stained agarose gels. The antibodies used were IgG1 (BD Biosciences, San Jose, CA), c-myc (9E10, Sigma-Aldrich, St. Louis, MO), E2F3 (C18, Santa Cruz Biotechnology, Santa Cruz, CA), ERα (Ab10, clone TE111.5D11; Thermo Scientific, Rockford, IL), histone H3 (ChIP grade; Abcam, Cambridge, MA), and SP1 (Millipore, Billerica, MA). The primers to amplify the *DEK* promoter at ChIP 2 are (F) AGG CAG CAT TGA GCA GGG AAG AT and (R) GGG TGT AAA TCT ATG TGG CT and at ChIP 4 are (F) GCG GTC CTC AGA AAT ACA GAT TTG GG and (R) GTG GAA ACA ATA AAC ACG CAG GCC. The primer sequences to amplify the *pS2* gene promoter are: (F) CCG GCC ATC TCT CAC TAT GAA and (R) CCT TCC CGC CAG GGT AAA TAC.

### Luciferase Assays

The first exon of the *DEK* gene and 1200 bp of the proximal promoter were inserted upstream of a luciferase reporter gene in the pGL3-basic plasmid. The *DEK* reporter construct and Renilla (pRL-TK) were transfected into MCF7 and T47D cells cultured in CS-FBS using Mirus TransIT-LT1 (Mirus Bio LLC, Madison, WI). Twenty-four hours post-transfection, cells were treated with vehicle (ethanol; EtOH), 17β-estradiol, or tamoxifen for an additional 24 hours then collected and analyzed using the Dual Luciferase Reporter assay system (Promega Corporation, Madison WI). Results represent fold-change compared to untreated (EtOH) control cells.

### Lentiviral Transduction

Cells were transduced with the lentiviral pLKO.1 constructs (Sigma Aldrich Mission shRNA library, Sigma-Aldrich, St. Louis, MO) and selected in puromycin. DEKsh2 represents construct pLKO.1_DEK832 (targeting DEK mRNA at nucleotide position 832). DEKsh2 functionality was published previously [7], [11].

### Flow Cytometry

Cells were labeled for BrdU incorporation and analyzed by flow cytometry according to manufacturer’s instructions (BD Biosciences, San Jose, CA). Cells were counter-stained with 7-amino-actinomycin D (7-AAD) to analyze sub-G1 content with a FACSCalibur flow cytometer (BD Biosciences, San Jose, CA). Cells were gated based on forward- and side-scatter to minimize cellular debris that could skew the results.

### Statistics

The χ^2^ test was used to identify correlations between DEK expression and clinico-pathological variables on the tissue microarray; otherwise, statistical significance was assayed using Student’s t-test or ANOVA, as indicated in the figure legends. All *in vitro* experiments represent the average of triplicate experiments and errors bars depict standard error. In the figures, one asterisk (*) indicates p<0.05, and two asterisks (**) indicates p<0.01.

## Supporting Information

Figure S1 **17β-estradiol treatment results in increased DEK expression and phosphorylation of p44/42 (Erk1/2).** MCF7 cells cultured under hormone starvation conditions were either untreated or treated for 24 hours with 10 nM 17β-estradiol. Whole cell lysates were subjected to western blotting and probed with antibodies for DEK, phospho-p44/42, total p44/42, and Actin.
(TIF)

Figure S2 **Dose response of DEK expression to 17β-estradiol treatment.** (A) Microarray studies using MCF7/BUS cells treated with varying concentrations of 17β-estradiol show that *DEK* expression is increased following treatment with 17β-estradiol. This data was originally published by Coser, K.R. *et al* [32]. (B) Western blotting of T47D cells grown in CS-FBS for seven days indicated that even 10 pM of 17β-estradiol for 48 hours is sufficient to stimulate DEK expression.
(TIF)

Figure S3 ***DEK*** **expression is transiently upregulated with 17β-estradiol treatment.** *DEK* expression increases rapidly in 17β-estradiol treated cells but returns to baseline levels upon prolonged exposure. Quantitative RT-PCR was performed to detect *DEK* expression in MCF7 cells grown in CS-FBS treated with 10 nM 17β-estradiol for 6, 24, and 48 hours. Expression was normalized to *GAPDH* transcript levels.
(TIF)

Figure S4 **DEK depletion is synergistic with tamoxifen treatment to induce apoptosis in the MCF7 breast cancer cell line.** (A) MCF7 cells transduced with non-targeting (NTsh) or DEK-targeting (DEKsh2) lentiviral shRNA constructs were grown in reduced serum and either untreated (EtOH) or treated with tamoxifen for 18 hours. Cells were fixed in 2% paraformaldehyde and stained with 4′,6-diamidino-2-phenylindole (DAPI). Apoptotic cells are indicated with white arrows and show the condensation and fragmentation of DNA. Percentages of apoptotic cells are shown in the bottom right corner of each image. (B) Western blotting of whole cell lysates from MCF7 NTsh and DEKsh2 cells indicates increased levels of cleaved caspase 8, a marker of apoptosis, in DEKsh2 cells treated with tamoxifen. Actin was used for normalization and the numbers below indicate the fold-change in cleaved caspase 8 levels, compared to untreated NTsh cells, as determined by densitometry.
(TIF)
