# Supplementary figures and images for: The DEK Oncogene Is a Target of Steroid Hormone Receptor Signaling in Breast Cancer
Source: PLoS One. 2012 Oct 10;7(10):e46985. doi: 10.1371/journal.pone.0046985 (PMC3468546; doi:10.1371/journal.pone.0046985)

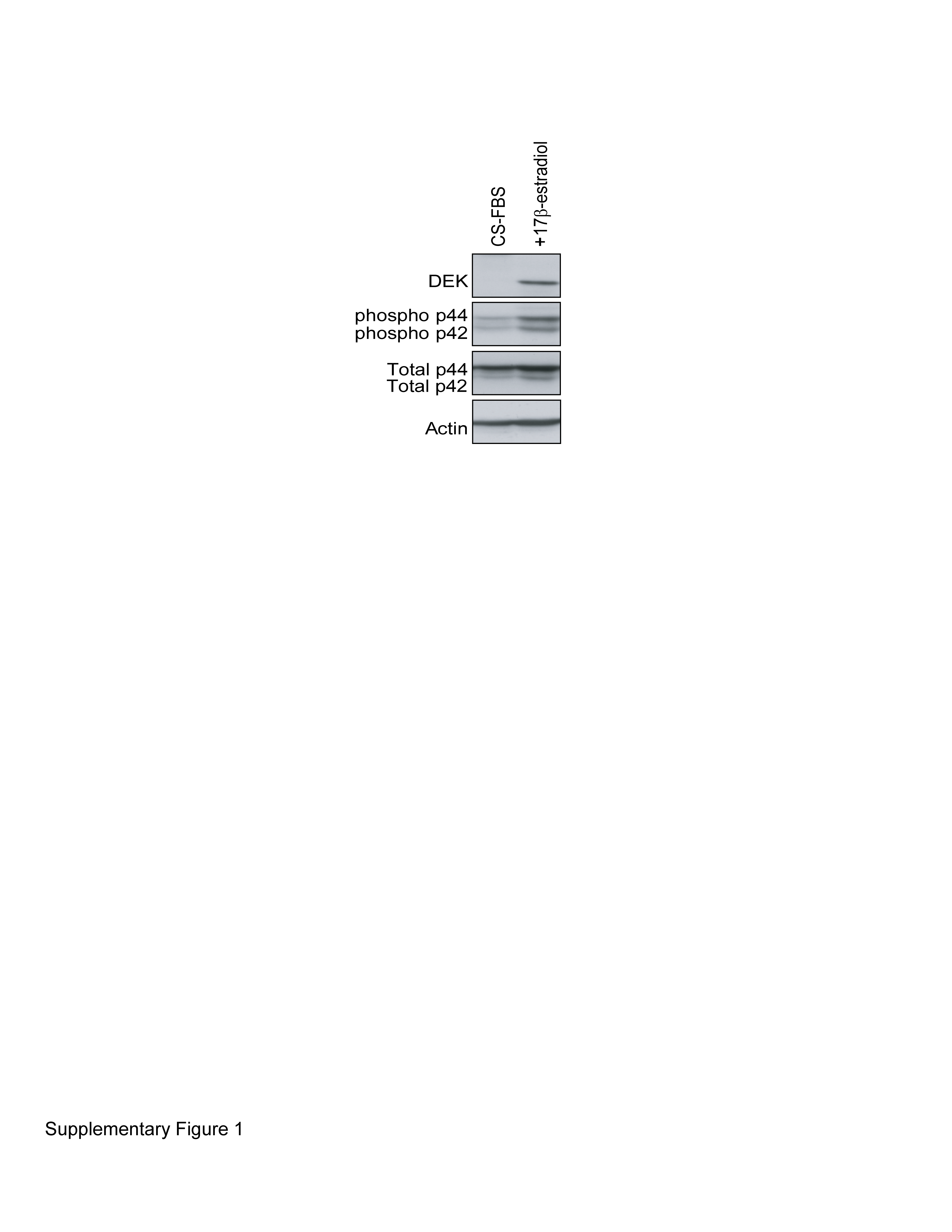

Supplement: Figure S1 — 17β-estradiol treatment results in increased DEK expression and phosphorylation of p44/42 (Erk1/2). MCF7 cells cultured under hormone starvation conditions were either untreated or treated for 24 hours with 10 nM 17β-estradiol. Whole cell lysates were subjected to western blotting and probed with antibodies for DEK, phospho-p44/42, total p44/42, and Actin. (TIF) [file pone.0046985.s001.tif]

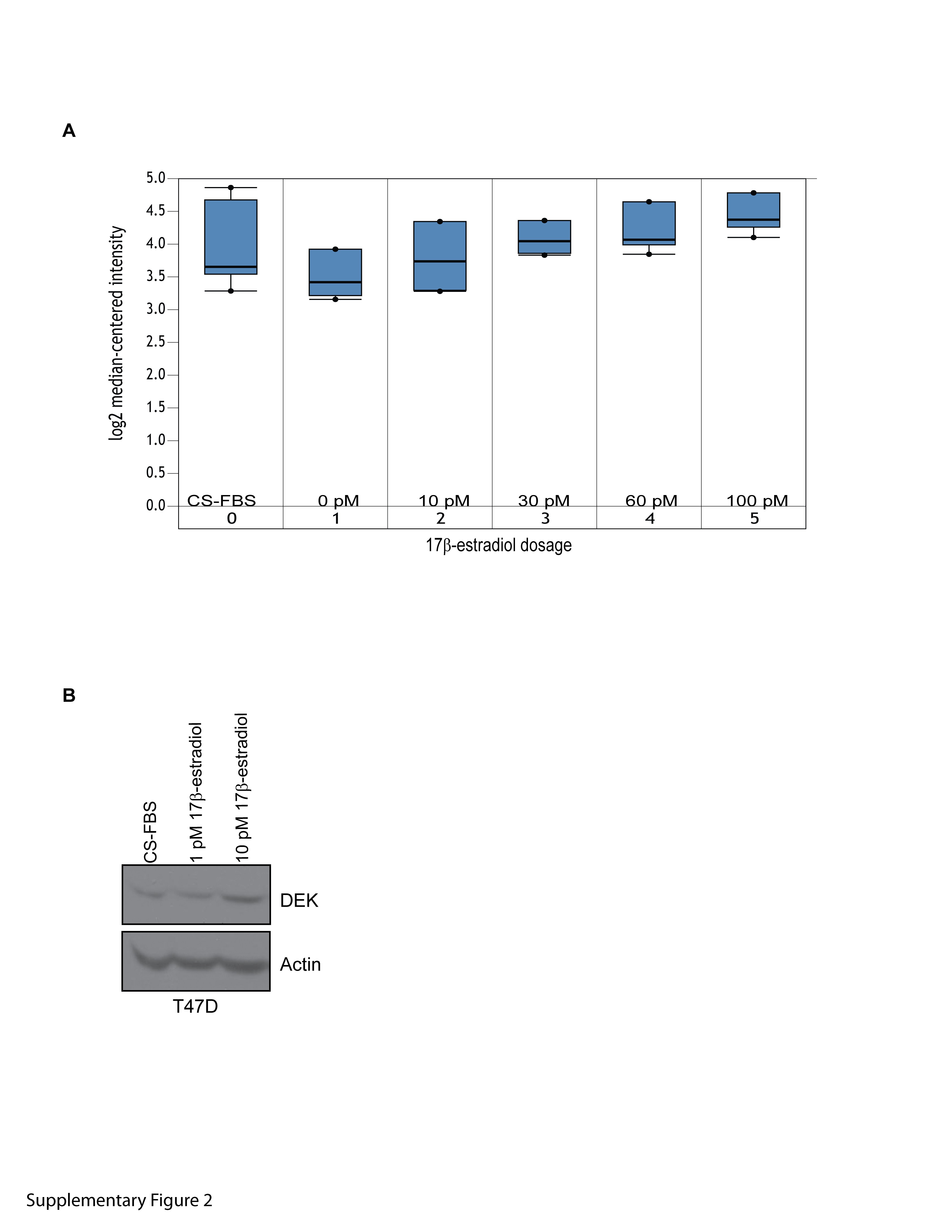

Supplement: Figure S2 — Dose response of DEK expression to 17β-estradiol treatment. (A) Microarray studies using MCF7/BUS cells treated with varying concentrations of 17β-estradiol show that DEK expression is increased following treatment with 17β-estradiol. This data was originally published by Coser, K.R. et al [32]. (B) Western blotting of T47D cells grown in CS-FBS for seven days indicated that even 10 pM of 17β-estradiol for 48 hours is sufficient to stimulate DEK expression. (TIF) [file pone.0046985.s002.tif]

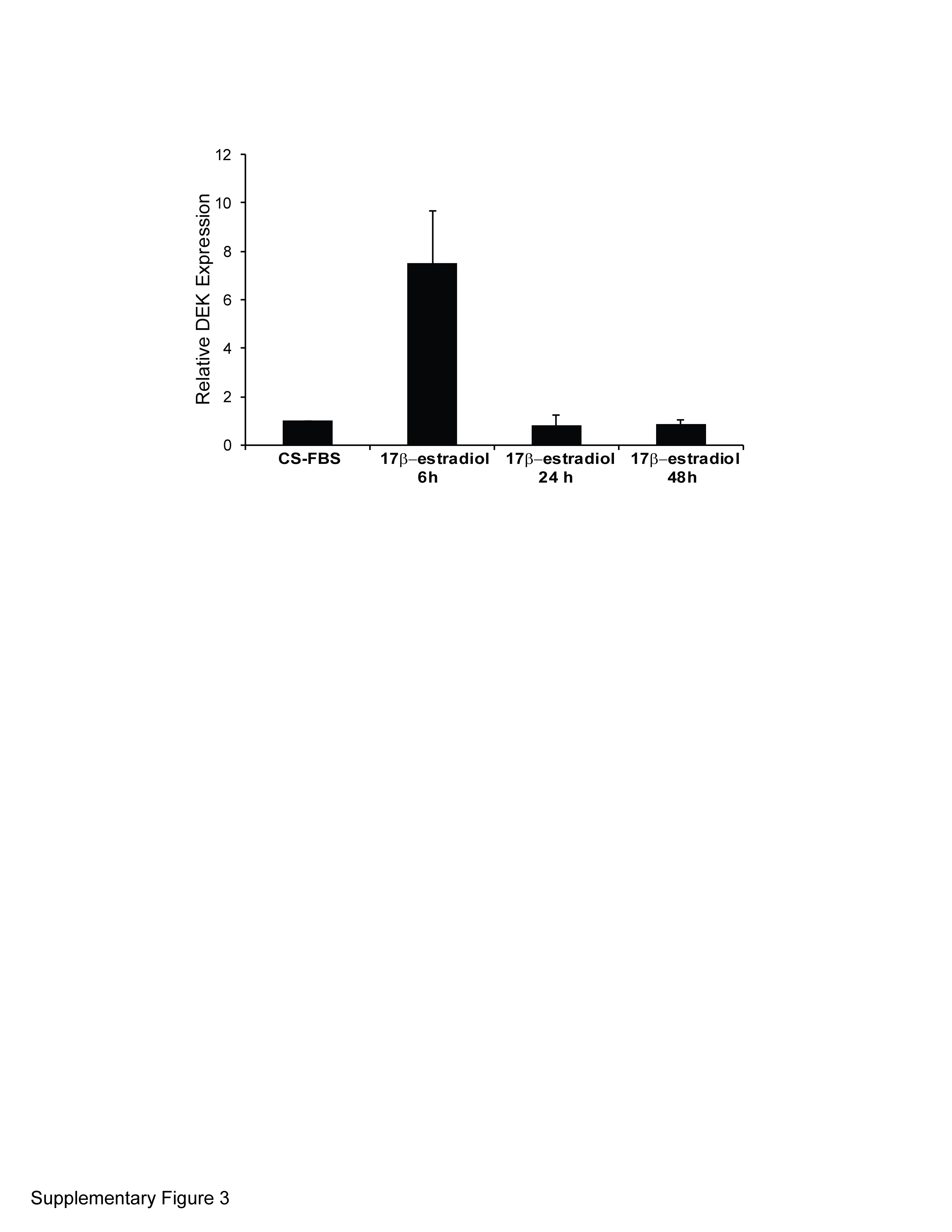

Supplement: Figure S3 — DEK expression is transiently upregulated with 17β-estradiol treatment. DEK expression increases rapidly in 17β-estradiol treated cells but returns to baseline levels upon prolonged exposure. Quantitative RT-PCR was performed to detect DEK expression in MCF7 cells grown in CS-FBS treated with 10 nM 17β-estradiol for 6, 24, and 48 hours. Expression was normalized to GAPDH transcript levels. (TIF) [file pone.0046985.s003.tif]

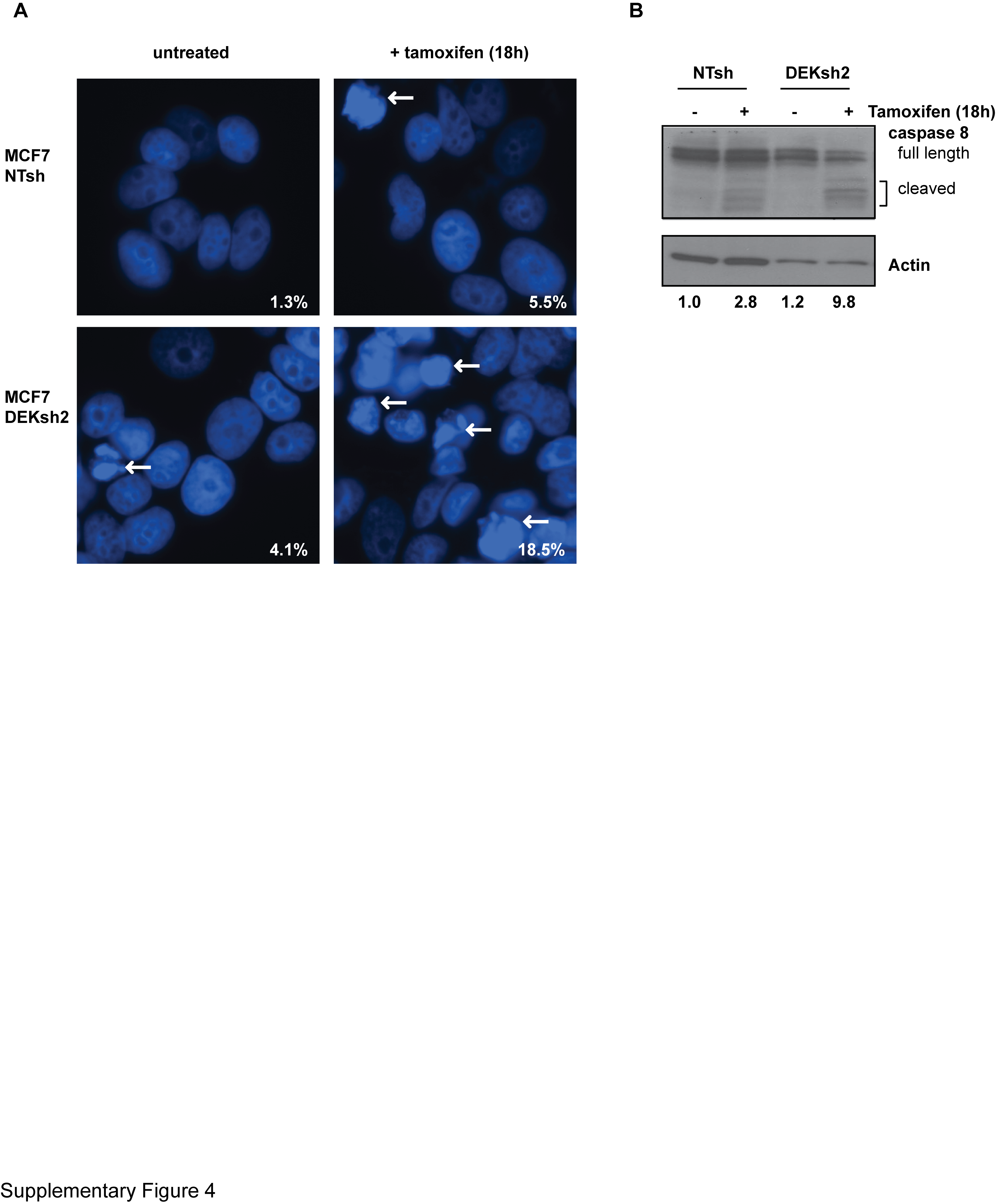

Supplement: Figure S4 — DEK depletion is synergistic with tamoxifen treatment to induce apoptosis in the MCF7 breast cancer cell line. (A) MCF7 cells transduced with non-targeting (NTsh) or DEK-targeting (DEKsh2) lentiviral shRNA constructs were grown in reduced serum and either untreated (EtOH) or treated with tamoxifen for 18 hours. Cells were fixed in 2% paraformaldehyde and stained with 4′,6-diamidino-2-phenylindole (DAPI). Apoptotic cells are indicated with white arrows and show the condensation and fragmentation of DNA. Percentages of apoptotic cells are shown in the bottom right corner of each image. (B) Western blotting of whole cell lysates from MCF7 NTsh and DEKsh2 cells indicates increased levels of cleaved caspase 8, a marker of apoptosis, in DEKsh2 cells treated with tamoxifen. Actin was used for normalization and the numbers below indicate the fold-change in cleaved caspase 8 levels, compared to untreated NTsh cells, as determined by densitometry. (TIF) [file pone.0046985.s004.tif]
